# Indirect-comparison meta-analysis of treatment options for patients with refractory Kawasaki disease

**DOI:** 10.1186/s12887-019-1504-9

**Published:** 2019-05-17

**Authors:** Han Chan, Huan Chi, Hui You, Mo Wang, Gaofu Zhang, Haiping Yang, Qiu Li

**Affiliations:** 1 Department of Nephrology, Children’s Hospital of Chongqing Medical University, Ministry of Education Key Laboratory of Child Development and Disorders, China International Science and Technology Cooperation base of Child Development and Critical, Chongqing, 400014 China; 2 Graduate School of Chongqing Medical University, Chongqing, 400016 China

**Keywords:** Mucocutaneous lymph node syndrome, Immunosuppressant, Intravenous immunoglobulin, Methylprednisolone, Infliximab, Second IVIG infusion

## Abstract

**Background:**

There is limited information available regarding the clinical management of intravenous immunoglobulin-resistant Kawasaki disease (KD). We aimed to evaluate the optimal treatment options for patients with refractory KD by presenting an indirect-comparison meta-analysis.

**Methods:**

PubMed, EMBASE, Web of Science, and the Cochrane Database were searched on August 31, 2018. Unpublished studies were also searched in ProQuest Dissertations & Theses and through manual retrieval strategies. Randomized concurrent controlled trials (RCTs), high-quality non-randomized concurrent controlled trials (non-RCTs), and retrospective studies associated with AEs were included. The quality of all eligible studies was assessed using Cochrane collaboration’s tool and non-randomized study guidelines. Risk ratios (RR) with 95% confidence intervals (CIs) for dichotomous outcomes were estimated in our analysis. GRADE profiler 3.6.1 was used to assess the evidence profile.

**Results:**

Twelve studies involving 372 immunoglobulin-resistant KD patients were identified and analyzed. Neither infliximab nor intravenous pulse methylprednisolone (IVMP) was significantly more effective than second IVIG infusion with respect to lowering coronary artery lesions (CALs) (infliximab, 0.85, 0.43–1.69; IVMP, 0.99, 0.52–1.88) and treatment resistance (infliximab, 0.43, 0.21–0.89; IVMP, 1.16, 0.33–4.13). No significant differences were found between infliximab and IVMP in the incidence rate of CALs (0.70, 0.27–1.81), the treatment resistance (0.37, 0.09–1.60), the rates of coronary artery aneurysm (4.13, 0.38–45.22) and the coronary artery dilatation (0.45, 0.10–1.99). Furthermore, compared with second IVIG infusion, both infliximab and IVMP showed significant effectiveness in antipyretic effects (infliximab, 1.52, 1.16–1.99; IVMP, 1.29, 0.77–2.15). However, Infliximab was noninferior to IVMP on antipyretic effects (1.18, 0.66–2.15). IVMP treatment showed significant association with fewer AEs than second IVIG infusion (0.49, 0.26–0.94) and infliximab (2.34, 1.07–5.09). No significant differences were noted between infliximab treatment and second IVIG infusion (1.06, 0.69–1.63).

**Conclusions:**

Infliximab, IVMP, and second IVIG infusion showed no significant differences in the cardioprotective effect or the rate of treatment resistance. Infliximab and IVMP treatment were more effective than second IVIG infusion regarding antipyretic effects. IVMP treatment may have an advantage due to its lower total rate of AEs associated with drug infusion.

**Trial registration:**

The study has been registered on PROSPERO (CRD42016039693).

**Electronic supplementary material:**

The online version of this article (10.1186/s12887-019-1504-9) contains supplementary material, which is available to authorized users.

## Background

Kawasaki disease (KD) is an acute self-limited systemic vasculitis that occurs mainly in infants and children [1]. KD involves multiple organs and tissues. Approximately fifteen to 25 % of untreated children with KD develop coronary artery lesions (CALs) or coronary artery aneurysms(CAA) [2]. CALs are associated with myocardial infarction, sudden death, and heart disease [3]. Relevant treatment in the acute phase is directed at reducing inflammation in the coronary artery wall and preventing CALs. Intravenous immunoglobulin (IVIG) is recognized as the first-line therapy for KD, and it has been shown to reduce the incidence of CALs. However, at least 10% of patients with KD fail to respond to initial IVIG treatment [4, 5], and second IVIG infusion (2 g/kg) has become a common practice. However, fever persists in approximately half of KD patients who receive a second IVIG dose, and this subset of patients has a higher risk than other subsets of developing CALs [6]. Therefore, the identification of additional potentially useful therapies for the treatment of immunoglobulin-resistant KD has become a focus of clinical trials [7].

Intravenous pulse methylprednisolone (IVMP, 30 mg/kg for 2 to 3 h once daily for 1 to 3 days) is the most commonly used steroid regimen, which rapidly inhibits inflammation and suppresses cytokine levels in KD patients. Several clinical trials have investigated the efficacy of steroids in IVIG nonresponders [8–12], but some were poor-quality randomized controlled trials (RCTs) or revealed controversial results. Thus far, the role of IVMP in the initial treatment of immunoglobulin-resistant KD patients has not been established. Infliximab is a chimeric monoclonal antibody against TNF-α under investigation in several clinical trials as a treatment for children who fail to respond to initial IVIG [13–15]. Similar to IVMP, infliximab is regarded as a new adjunctive therapy that may have positive effects in the treatment of patients with acute KD [16].

Currently, infliximab, IVMP, and second IVIG infusion are the conventional care for immunoglobulin-resistant KD patients who have failed the initial standard therapy. However, the efficacy of and adverse effects (AEs) associated with these drug administrations are not well known. In the absence of any trials directly assessing the efficacy and AEs of infliximab and methylprednisolone treatment for immunoglobulin-resistant KD, one method to evaluate efficacy and AEs is to conduct an adjusted indirect comparison of data from existing trials with a common control [17].

An indirect comparison is an ideal method by which to resolve issues when there is no direct evidence from current clinical trials. If direct evidence of both α versus γ and β versus γ is available, an indirect comparison of α versus β is conducted using the same intervention γ as a common comparator. The meta-analysis defined second IVIG infusion as the common comparator. This adjusted indirect comparison meta-analysis aimed to evaluate the safety and effectiveness of these three therapies for children with immunoglobulin-resistant KD in the hope of providing evidence-based clinical advice.

## Methods

Ethical approval was not required because this was a meta-analysis of previously published trials and no real patients were included. The meta-analysis conformed to standard guidelines and was written according to the PRISMA statement [18]. This review also follows a published protocol [19].

### Data collection and analysis

#### Database search strategy

We searched PubMed, EMBASE, Web of Science, and the Cochrane Database for articles published from each database’s date of inception to August 31, 2018, using a combination of basic text and MeSH terms. Specifically, we performed a MeSH search using ‘mucocutaneous lymph node syndrome’ and a keyword search using the phrase ‘Kawasaki disease’ and terms related to intravenous immunoglobulin (including a MeSH search using ‘immunoglobulins, intravenous’ and a keyword search using the words ‘intravenous immunoglobulin’, ‘intravenous gamma globulin’, ‘IVIG’, ‘IVGG’, and ‘IG’). This search strategy was modified to fit each database. In addition, unpublished studies were searched in ProQuest Dissertations & Theses and following manual retrieval strategies; we reviewed (1) references from published articles to identify additional relevant studies, (2) conference proceedings likely to contain trials relevant to the analysis, and (3) unpublished data or incomplete trials for relevant trial authors. All searches included non-English language literature (for the full search strategy, see Additional file 1).

#### Selection criteria and process

The following studies were included in the meta-analysis: (1) RCTs and high-quality non-randomized concurrent controlled trials (non-RCTs); retrospective studies (e.g., cohort studies or case-control studies) associated with AEs were reviewed; (2) studies whose patient populations included children with immunoglobulin-resistant KD according to the criteria of the Japanese Ministry of Health and Welfare [20] or the American Heart Association (AHA) [1], which defines immunoglobulin-resistant KD as KD characterized by persistent or recrudescent fever lasting longer than the specified observation period (24, 36, or 36–48 h) after completion of an initial IVIG infusion; (3) studies including patients considered to have diseases complicated by CALs (i.e., patients with dilatations or aneurysms of varying severity according to the classical criteria of the Japanese Ministry of Health and Welfare or AHA); (4) studies including patients who received infliximab or IVMP treatment for immunoglobulin-resistant KD after failing initial IVIG therapy; (5) treatment resistance was defined as the need for further treatment after completing infliximab or IVMP treatment. Antipyretic effects were defined as fever resolution or a significant decrease in a persistent fever within 3 days of completing the drug infusion without another explanation; and (6) studies with baseline patient demographics, disease characteristics, laboratory data, and CALs incidence that were similar between the two groups. Studies that failed to meet the inclusion criteria were excluded from the analysis.

#### Data collection and outcome measures

Studies were selected by 2 independent reviewers (H. You and H. Chi) according to the above inclusion criteria, and disputes regarding the studies were resolved by H. Chan. Data extracted from each study included the publication year, age, setting, design, number of cases, initial course of the disease, initial treatment, retreatment, and the follow-up time points at which echocardiographic assessments were performed. The primary outcomes were CALs and the rate of treatment resistance. The secondary outcomes were AEs associated with drug infusion and antipyretic effects.

#### Assessment of the risk of bias in included studies

The methodological quality of the included RCTs was assessed using the Cochrane collaboration tool to assess the risk of bias [21]; The Methodological Index for Non-Randomized Studies (MINORS) guidelines were selected to assess the methodological quality of the non-RCTs [22]. A quality assessment of the studies was performed using the NOS (http://www.ohri.ca/programs/clinical_epidemiology/oxford.asp) under the three main categories [23].The overall quality of the evidence and strength of the recommendations were evaluated using the GRADE system [24].

### Statistical analysis

A traditional pair-wise meta-analysis was conducted. All statistical analyses were performed using Stata 14.0 software (Stata Corp., College Station, TX, USA) [25]. The risk of bias assessment was performed with Review Manager 5.3 software (The Nordic Cochrane Centre, Copenhagen, Denmark), in accordance with the guidelines outlined in the Cochrane Handbook (version 5.1.0) [21]. We estimated the Risk ratios (RR) and 95% confidence interval (CI) for dichotomous outcomes and used a random-effects model regardless of the presence of heterogeneity. Sensitivity analyses were performed to evaluate the effect of each study on the pooled RR. Between-study heterogeneity was tested using the *I*^*2*^ test and considered significant at *I*^*2*^ > 50% or *P* < 0.1. GRADE profiler 3.6.1 was used to assess the evidence profile [26].

## Results

### Study selection and description

**Fig. 1:**
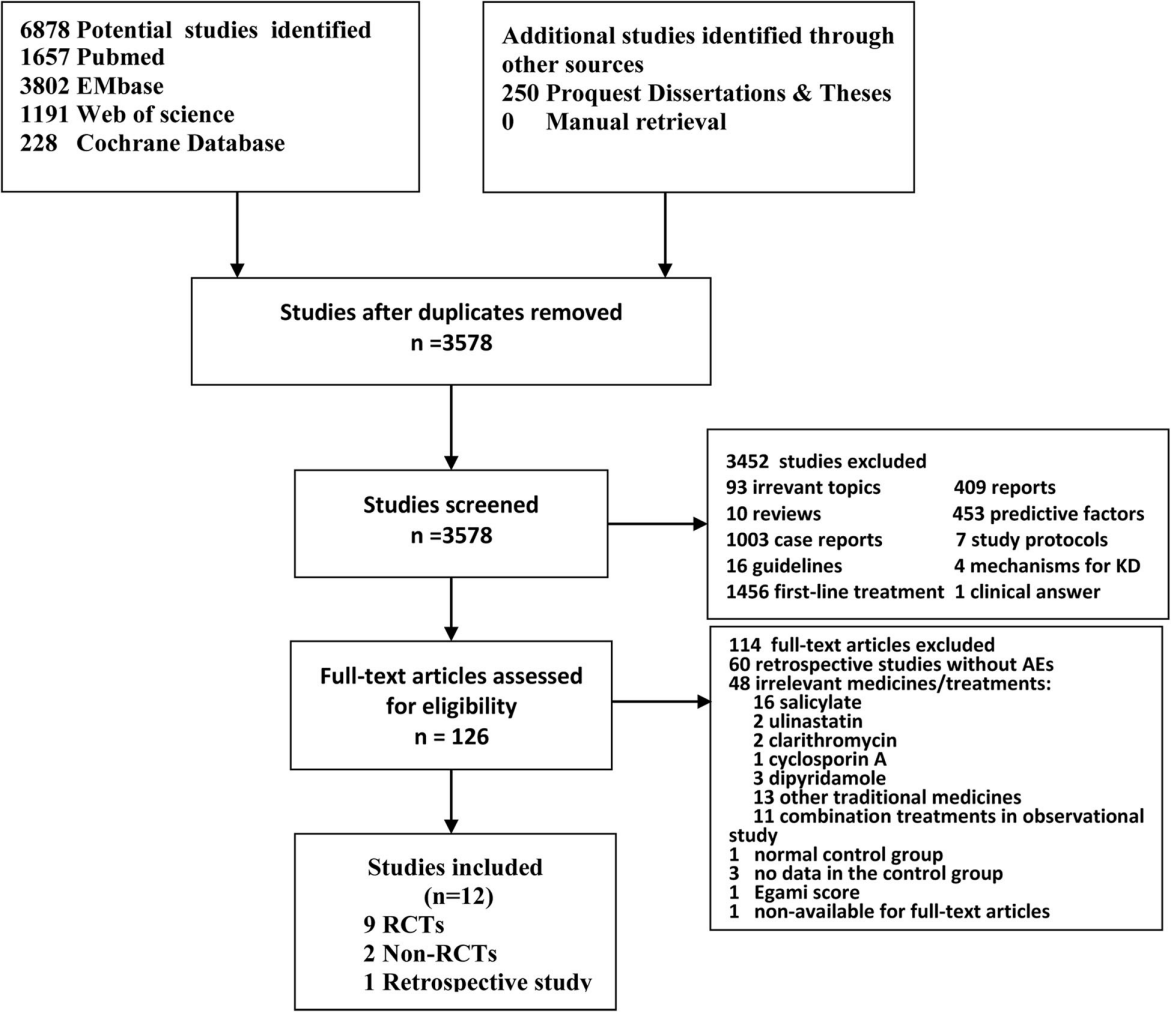
Flow diagram for selection of trials and reasons for study exclusion

We initially identified 7128 potentially relevant studies (Fig. 1). A total of 12 studies, being published between 2003 and 2018, were included in the meta-analysis according to the inclusion and exclusion criteria**,** of which, nine studies were RCTs, while remaining trials were non-RCTs, according to the Cochrane Handbook. Those two non-RCTs trials (Furakawa et al. [9] and Teraguchi et al. [12]) did not adopt random sequence generation because a portion of their patients refused IVMP treatment and were treated with second IVIG infusion instead. Neither the blinding method nor the allocation concealment method was mentioned in the reports of the non-RCTs. In addition, six studies defined the body temperature being over 38 °C as a symbol of recurrent or persistent fever of non-responsiveness in KD patients while two other studies each adopted 37.5 °C and 38.3 °C, and this cut off value was not recorded in two other studies. Six studies defined 36 h as the observation period after drug infusion, five studies used 36–48 h, and one study used 24 h. Treatment groups had similar baseline characteristics at admission, including sex, ethnicity, age at fever onset, time from fever onset to diagnosis, and time from first treatment to retreatment. Discrepant inflammation intensity between treatment groups was reported in many of the selected studies but not in Youn et al. [15], Tremoulet et al. [16], Son et al. [27], Furakawa et al. [9], Masaaki et al. [28], and Newburger et al. [29].

Overall, the 12 selected studies included 372 patients with immunoglobulin-resistant KD after removing the ineligible patients in the studies by Tremoulet et al. [16], Sundel et al. [30] and Newburger et al. [29]. The follow-up time point at which echocardiography was performed ranged from study entry to 7 months post-treatment in the infliximab group. In the IVMP treatment group, the follow-up time point ranged from study entry to 6 weeks post-treatment. Table 1 presents the characteristics of included studies.

**Table 1:** Characteristics of the trials included in the analysis

Author	Year	Patient age (treatment/control)	Setting	Design	Cases	Disease course	Initial treatment^a^ (treatment/control)	Retreatment^a^ (treatment/control)	Follow-up	NOS /MINORS
Burns et al. [14]	2008	22**/**20 months	US	RCT	12/12	2–7 days	IVIG 2	Infliximab 5/IVIG 2	Study entry 1–2 weeks 6–8 weeks < 7 months	–
Youn et al. [15]	2016	3 months-13 years	Korea	RCT	11/32	3–8 days	IVIG 2	Infliximab 5/IVIG 2	Study entry 2–4 weeks	–
Tremoulet et al. [16]	2014	3.0/2.8 years	US	RCT	98/98	3–10 days	IVIG 2 + Infliximab 5 IVIG 2 + Normal saline	IVIG 2	Study entry 2–5 weeks	–
Son et al. [27]	2011	23/29 months	US	Retrospective	20/86	4–7 days	IVIG 2	Infliximab 5/IVIG 2	Study entry 1–10 weeks	8
Masaaki et al. [28]	2018	2.5/3.0 years	Japan	RCT	16/15	6-7 days	IVIG 2	Infliximab 5	Study entry 8 week	–
Miura et al. [8]	2005	N/A	Japan	RCT	11/11	N/A	IVIG 2	IVMP 30 for 3 consecutive days/IVIG 2	Study entry 1 week	–
Furakawa et al. [9]	2007	31.3/28.1 months	Japan	Non-RCT	44/19	N/A	IVIG 2	IVMP 30 for 3 consecutive days/IVIG 2	4 weeks	19
Miura et al. [10]	2008	32 ± 19/32 ± 26 months	Japan	RCT	7/8	4–5 days	IVIG 2	IVMP 30 for 3 consecutive days/IVIG 2	Study entry 1 week	–
Ogata et al. [11]	2009	14 ± 17/33 ± 24 months	Japan	RCT	13/14	4–5 days	IVIG 2	IVMP 30 for 3 consecutive days/IVIG 2	Before discharge	–
Teraguchi et al. [12]	2013	1–120 months	Japan	Non-RCT	14/27	N/A	IVIG 2	IVMP 30 for 3 consecutive days	4 weeks	20
Sundel et al. [30]	2003	4.3/4.5 years	US	RCT	18/21	6.5/6.9 days	IVIG 2 + IVMP 30/IVIG 2	N/A	2 and 6 weeks	–
Newburger et al. [29]	2007	2.9/2.9 years	US	RCT	101/97	4–10 days	IVIG 2 + IVMP 30/IVIG 2	IVIG 2	Study entry 1 and 5 weeks	–

^a^IVIG g kg^−1^ day^− 1^, IVMP mg kg^− 1^ day^− 1^, Infliximab mg kg^− 1^ day^− 1^, N/A Not available

### Risk of bias of included studies

**Fig. 2:**
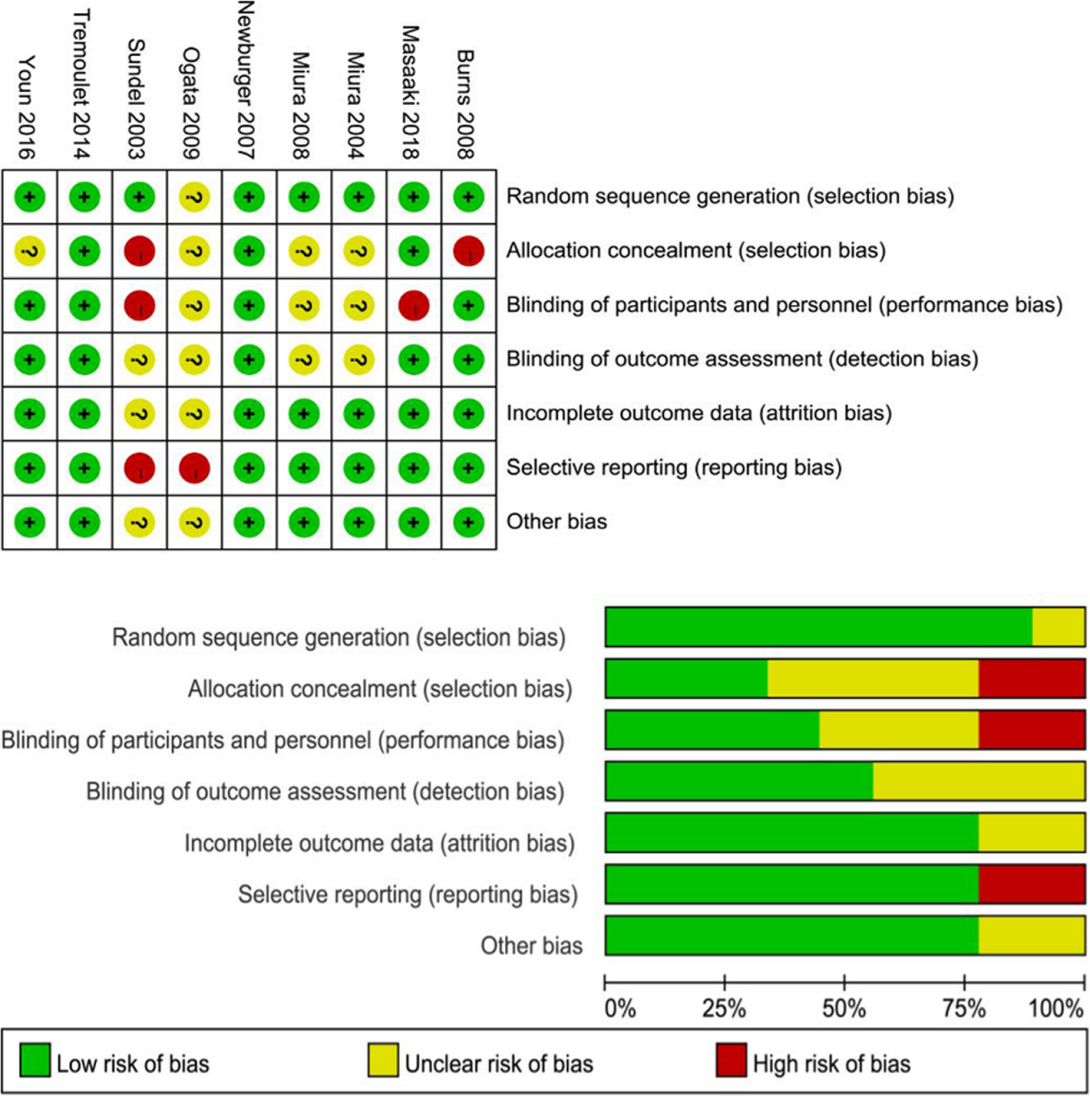
Assessment of the risk of bias

Two non-RCTs had scores ranging from 19 to 20 points according to the MINORS guidelines (Table 1), and both of these non-RCTs were marked as high quality. In accordance with the Newcastle-Ottawa scale (NOS) scale, the retrospective study scored 8 points and was judged to be of high relative quality (Fig. 2). Compared with the infliximab trials, the IVMP trials were of relatively low quality. Additionally, the risk of bias assessed by the Cochrane collaboration tool was higher in the IVMP trials than in the infliximab trials because the IVMP trials discussed only randomization without providing information regarding allocation concealment or blinded measurements, which might indicate a possible source of selection and performance bias (Fig. 2).

### Primary outcome

#### CALs

**Fig. 3:**
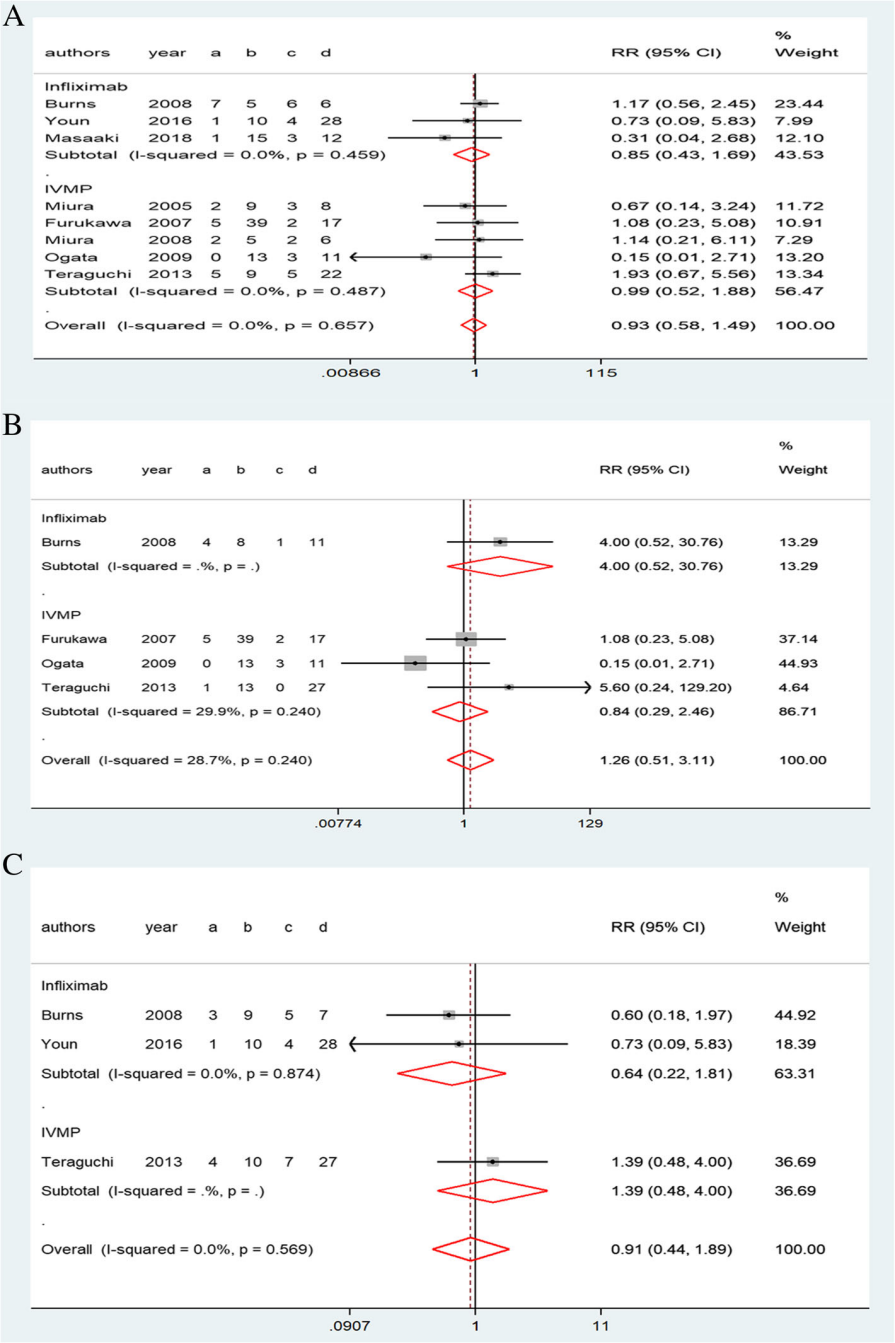
Forest plots of a traditional pair-wise meta-analysis of CALs in patients with immunoglobulin-resistant KD: (**a**) total incidence rate of CALs, (**b**) incidence of coronary artery aneurysms, and (**c**) incidence of coronary artery dilatation

Neither infliximab nor IVMP treatment was significantly more beneficial than second IVIG infusion with respect to reducing the total incidence rate of CALs in patients with immunoglobulin-resistant KD (infliximab, 0.85, 0.43–1.69, *P* = 0.46; IVMP, 0.99, 0.52–1.88, *P* = 0.49; Fig. 3a). No significant differences in the risk of a coronary artery aneurysm (infliximab, 4.00, 0.52–30.76; IVMP, 0.84, 0.29–2.46, *P* = 0.24; Fig. 3b) or coronary artery dilatation (infliximab, 0.64, 0.22–1.81, *P* = 0.87; IVMP, 1.39, 0.48–4.00; Fig. 3c) were found between the two treatment groups. No significant heterogeneity was observed among the studies (*I*^*2*^ = 0%). The indirect comparison relative risk (RR) of the total incidence rate of CALs for infliximab versus IVMP was 0.70(0.27–1.81, *P* = 0.46). No significant difference between infliximab and IVMP was found in the rate of coronary artery aneurysm (4.13, 0.38–45.22, *P* = 0.25) or coronary artery dilatation (0.45, 0.10–1.99, *P* = 0.29).

#### Treatment resistance

**Fig. 4:**
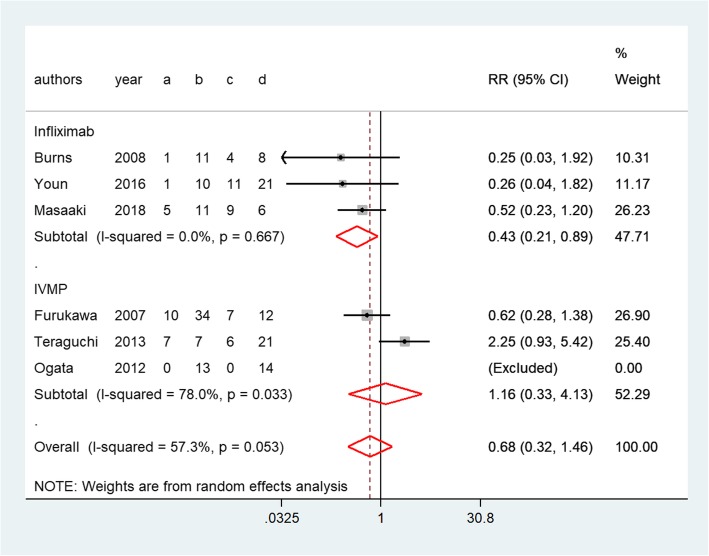
Forest plots of a traditional pair-wise meta-analysis of the rate of treatment resistance in patients with immunoglobulin-resistant KD

The rate of treatment resistance was not higher in the infliximab group than in the second IVIG infusion group (0.43, 0.21–0.89, *P* = 0.68, Fig. 4). Similarly, the meta-analysis showed that IVMP did not provide significantly more benefit than second IVIG infusion with respect to the rate of treatment resistance (1.16, 0.33–4.13, *P* = 0.03, Fig. 4). The indirect comparison RR of the rate of treatment resistance for infliximab versus IVMP was 0.37 (0.09–1.60, *P* = 0.18). Therefore, no significant difference in treatment resistance was found between infliximab and IVMP.

### Secondary outcomes

#### Antipyretic effects

**Fig. 5:**
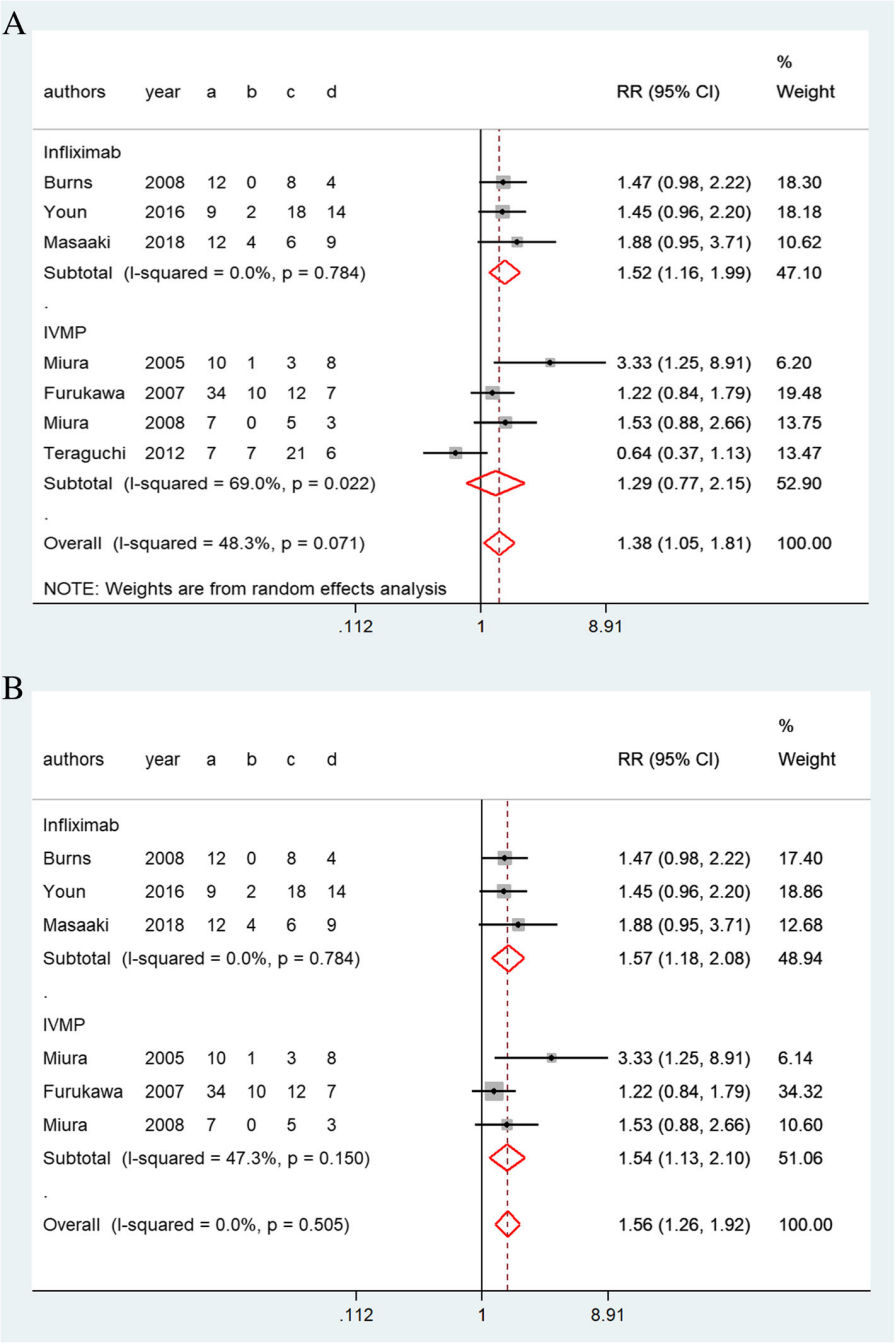
Forest plots of a traditional pair-wise meta-analysis of the antipyretic effects in patients with immunoglobulin-resistant KD: (**a**) before and (**b**) after sensitivity analysis

Infliximab was associated with significant antipyretic effects than second IVIG infusion (1.52, 1.16–1.99, *P* = 0.78, Fig. 5a). However, no significant differences were recorded between the IVMP group and the IVIG retreatment group (1.29, 0.77–2.15, *P* = 0.02, Fig. 5a) concerning the high level of heterogeneity (*I*^*2*^ = 69.0%). The indirect comparison RR of antipyretic effects for infliximab versus IVMP was 1.18 (0.66–2.15, *P* = 0.58), indicating that the antipyretic effects of infliximab and IVMP were not significantly different.

#### AEs

**Fig. 6:**
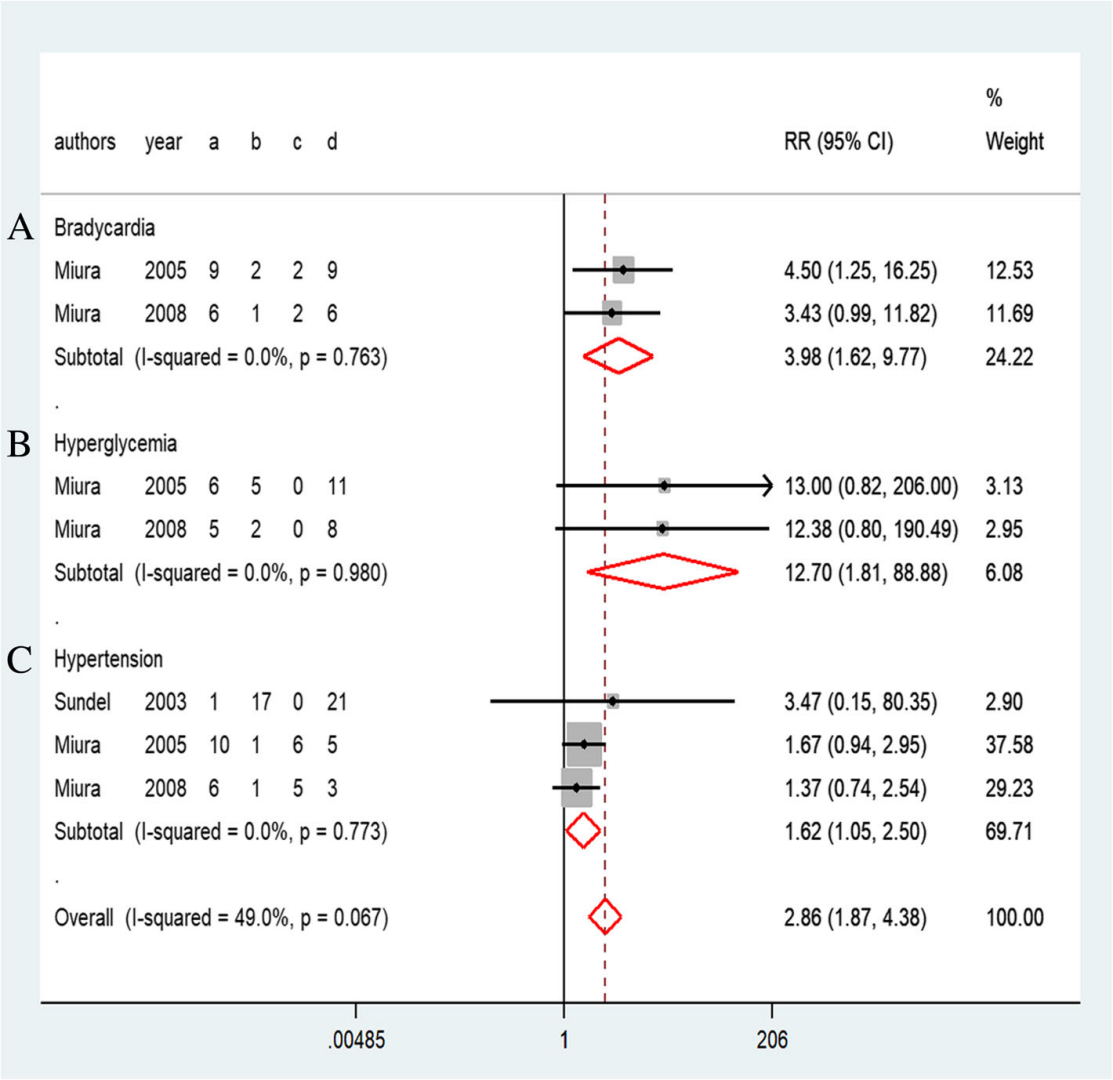
Forest plots of a traditional pair-wise meta-analysis of variable AEs during IVMP treatment: (**a**) bradycardia, (**b**) hyperglycemia, and (**c**) hypertension

**Fig. 7:**
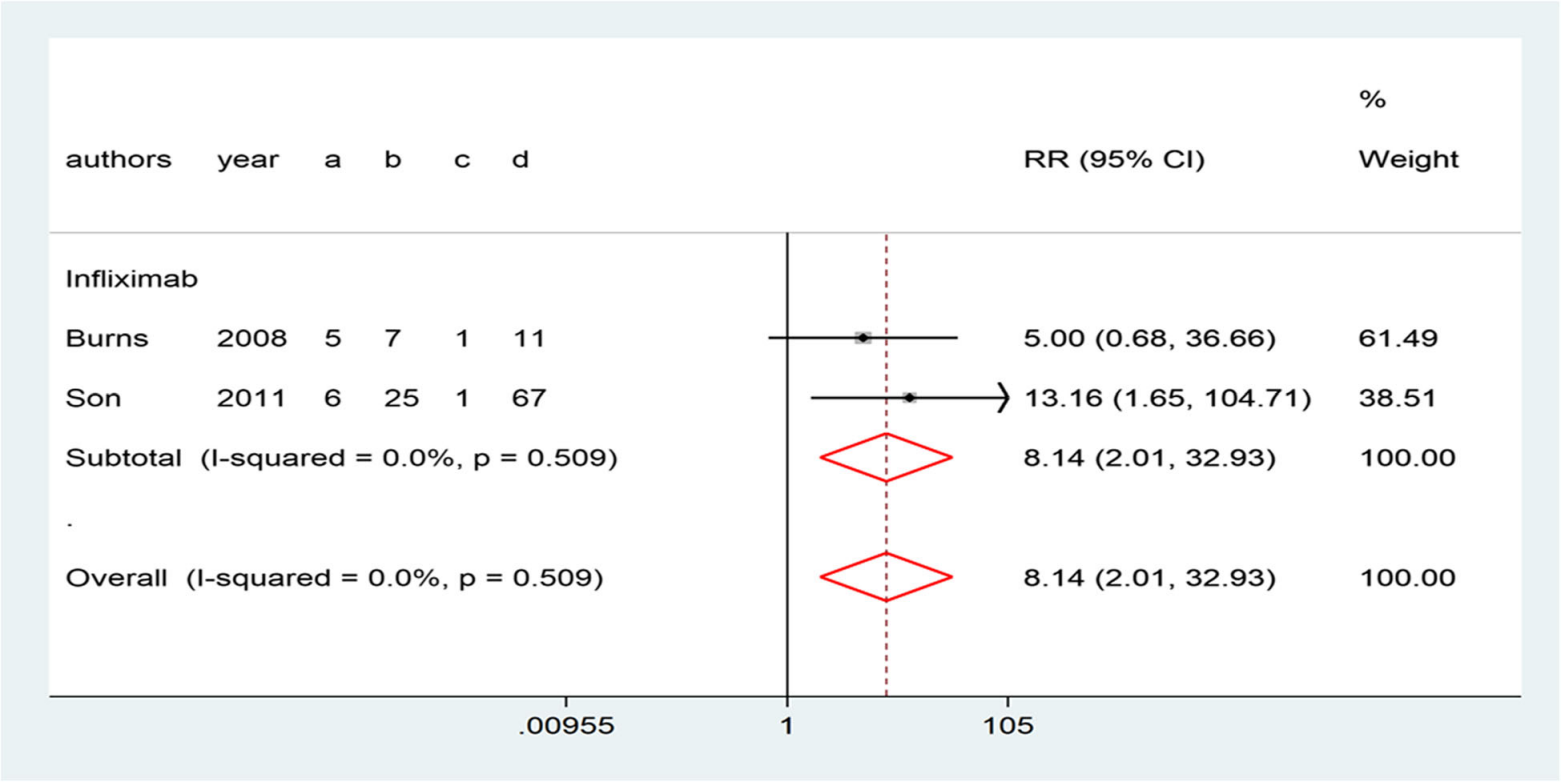
Forest plots of a traditional pair-wise meta-analysis of transient hepatomegaly during infliximab treatment

**Fig. 8:**
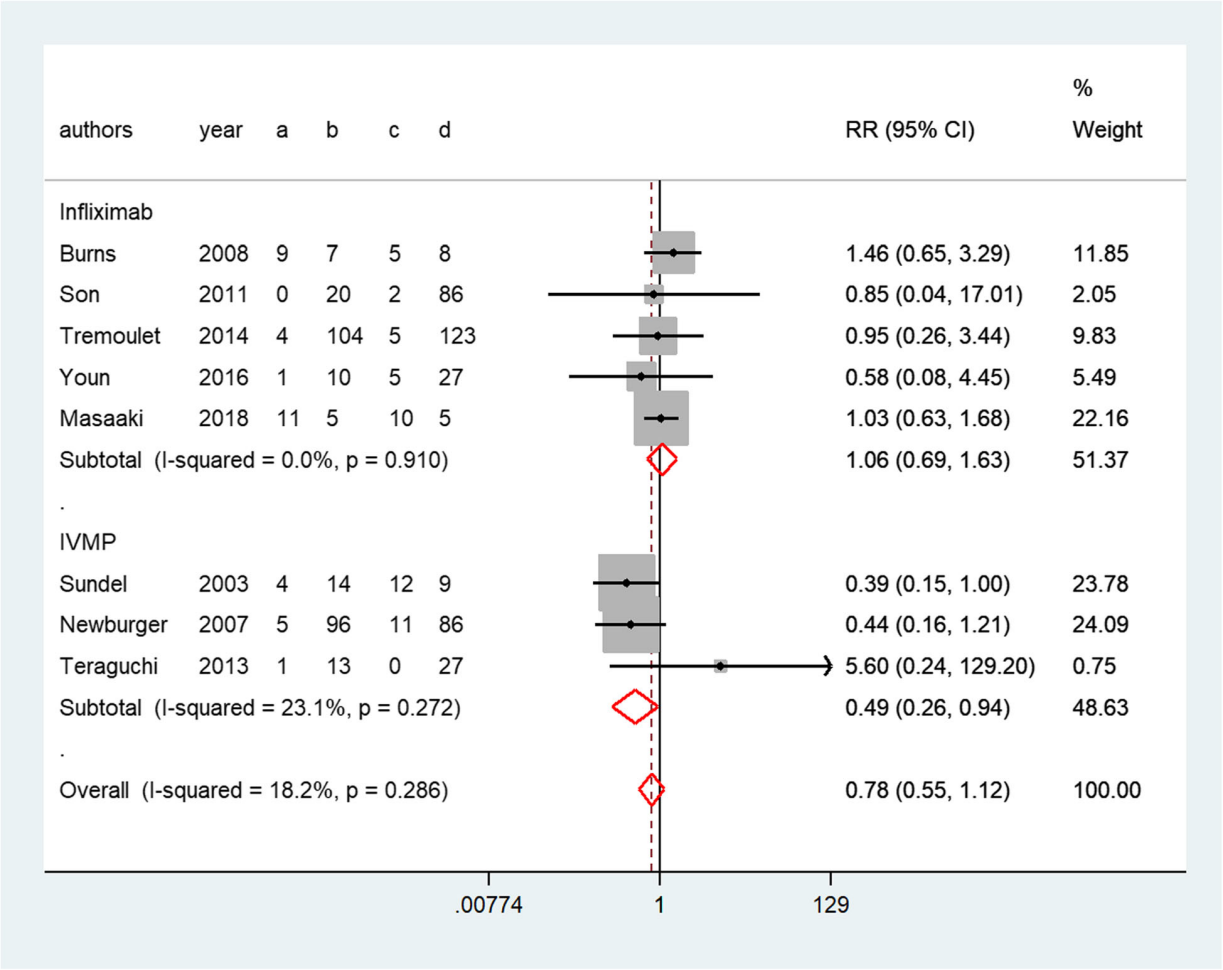
Forest plots of a traditional pair-wise meta-analysis of the total rate of adverse events associated with drug infusion in patients with immunoglobulin-resistant KD

Ten studies reported all AEs during the disease course [8, 10, 12, 14–16, 27–30]. In addition, eight of the studies reported AEs associated with infliximab or IVMP (Fig. 8). In summary, more AEs were reported in the IVMP group (Table 2), particularly bradycardia (3.98, 1.62–9.77, *P* = 0.76, Fig. 6), hyperglycemia (12.70, 1.81–88.88, *P* = 0.98, Fig. 6), and hypertension (1.62, 1.05–2.50, *P* = 0.77, Fig. 6). Conversely, patients undergoing infliximab treatment were more likely to suffer from transient hepatomegaly (8.14, 2.01–32.93, *P* = 0.50, Fig. 7).

**Table 2:** The incidence of AEs in the included studies

	Infliximab group (%)					IVMP group (%)				
Variable	Burns	Youn	Tremoulet	Son	Masaaki	Miura05	Miura08	Teraguchi	Sundel	Newburger
Transient hepatomegaly	41.7	–	–	19		–	–	–	–	–
Chills	–	0	–	–		–	–	–	6	–
Rash	–	10	1	–	12.5	–	–	–	–	–
Headache	–	–	2	–		–	–	–	–	5
Hemolytic anemia	–	–	2	–		–	–	–	–	1
Seizure	–	–	0	–		–	–	–	–	–
Hepatitis	–	–	0	–		–	–	–	–	–
Hypertension	–	–	0	–		90	86	–	6	–
Coagulopathy	–	–	0	–		27	–	–	–	–
Bradycardia	–	–	–	–		82	86	–	–	–
Hypothermia	–	–	–	–		1	14	–	–	–
Hypotension	–	–	–	–		–	–	–	–	5
Hyperglycemia	–	–	–	–		55	71	–	–	–
Embolism	–	–	–	–		0	0	–	–	–
Stool blood	–	–	–	–		0	0	7	–	–
Vomiting	–	–	–	–	6.3	–	–	–	0	–
Heart failure	–	–	–	–		–	–	–	0	0
Shock	–	–	–	–		–	–	–	–	1

Compared to second IVIG infusion, IVMP treatment was associated with fewer AEs (0.49, 0.26–0.94, *P* = 0.50, Fig. 8). Nevertheless, no significant differences were noted between infliximab treatment and second IVIG infusion (1.06, 0.69–1.63, *P* = 0.91, Fig. 8). Additionally, no significant heterogeneity was observed among the studies (*I*^*2*^ = 0%). The indirect comparison RR of the total rate of AEs for infliximab versus IVMP was 2.34(1.07–5.09, *P* = 0.03). The result showed that the total rate of AEs associated with drug infusion was lower for IVMP treatment than for infliximab treatment.

### Sensitivity analysis and GRADE evidence profile

**Fig. 9:**
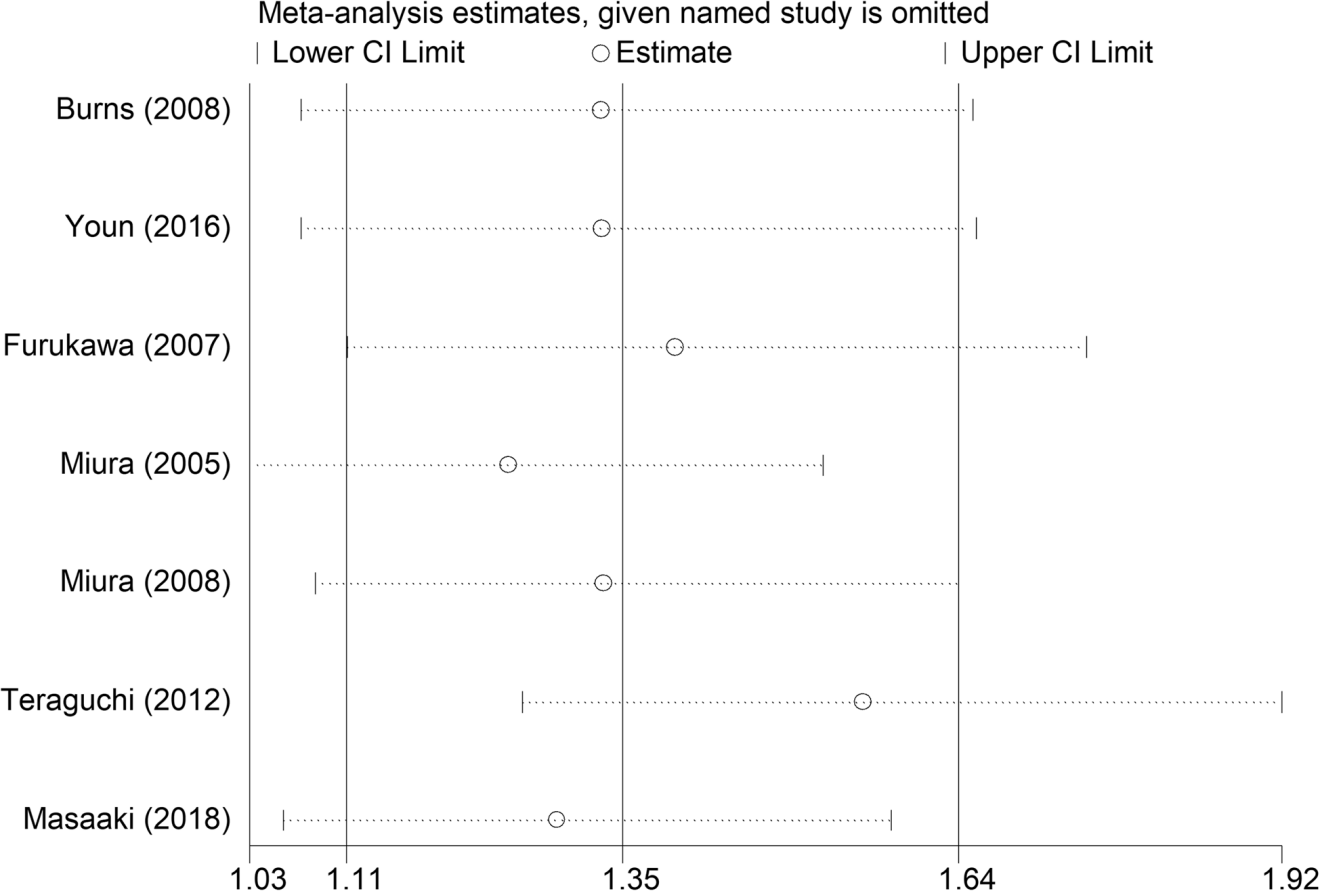
Sensitivity analysis

Significant heterogeneity was observed among the included studies in terms of antipyretic effects (*I*^*2*^ = 69%) and the rate of treatment resistance (*I*^*2*^ = 78%) in the IVMP treatment group. As shown in Figs. 5a and 9, the data reported in the study conducted by Teraguchi et al. [12] were completely out of range of those reported in other studies and probably contributed to the heterogeneity. The heterogeneity vanished after excluding this study (Fig. 5b). No evidence of heterogeneity was detected among the remaining studies.

Strengthened by the GRADE system, the working group grades of evidence were high for CALs in the infliximab group, moderate for CALs in the IVMP group, high for the rate of treatment resistance in the infliximab group, moderate for the rate of treatment resistance in the IVMP group, moderate for antipyretic action in both groups, moderate for the total rate of AEs associated with drug infusion in the infliximab group, and low for the total rate of AEs associated with drug infusion in the IVMP group. The indirect comparison suggested evidence grades of moderate for CALs between infliximab versus IVMP, low for treatment resistance between infliximab versus IVMP, moderate for antipyretic action between infliximab versus IVMP, and low for AEs associated with drug infusion among infliximab, IVMP, and second IVIG infusion [26].

### Publication bias

Tests for funnel plot asymmetry and meta-regression analyses weren’t conducted in the meta-analysis since the number of included studies in pair-wise meta-analysis was < 10, according to Cochrane Handbook [21].

## Discussion

TNF-α is elevated in the acute phase of KD and may be a contributing factor in patients who subsequently develop a coronary artery aneurysm. Infliximab, which is a chimeric monoclonal antibody against TNF-α, has been used to treat patients with immunoglobulin-resistant KD for the past 10 years. Several studies have suggested that treatment with infliximab results in faster fever resolution, shorter hospitalization, and even improved coronary artery outcomes compared to second IVIG infusion [15, 27] and that further treatment with infliximab may be an effective option for immunoglobulin/glucocorticoid-resistant KD patients with encephalitis [31]. However, the lack of sufficient clinical trials regarding this topic, as well as the small number of subjects included in the available trials, may have led to bias. A meta-analysis in 2017 [32] that included only 4 studies (2 RCTs of immunoglobulin-resistant KD patients, 1 RCT of initial treatment for KD patients, and 1 case-control study) showed that with the exception of antipyretic action, infliximab did not provide significantly more benefit than second IVIG with respect to the cardioprotective effect, rate of treatment resistance, and total rate of AEs. The authors took full advantage of the limited literature in this meta-analysis and merged data from studies with different designs. Our meta-analysis adopted a strict published protocol [19]. To acquire a reliable conclusion on the drug management of immunoglobulin-resistant KD patients, our meta-analysis not only analyzed infliximab treatment but also focused on an indirect comparison with IVMP treatment. All the outcomes except for AEs were measured predominantly using data from RCTs.

A previous traditional pair-wise meta-analysis of IVMP was published by Yang et al. in 2015 [33]. This analysis included only 4 studies involving a total of 52 patients and showed that IVMP was more effective than second IVIG infusion in controlling body temperature. Specifically, our subgroup analysis showed that IVMP was a more effective antipyretic than second IVIG infusion and that there was no significant difference in the overall incidence of CALs between IVMP and second IVIG. However, we regarded 2nd-line treatments merely as our endpoint; therefore, other drugs (3rd-line treatments) did not affect the realistic incidence of CALs and enabled us to avoid potential reporting bias. Furthermore, the fixed-effects model was not appropriate for the complex moderators in Yang’s work [34, 35]. Our meta-analysis further adopted the GRADE system and included an additional 8 studies (245 additional patients) to acquire more reliable clinical outcomes.

Our meta-analysis suggested that IVMP and infliximab may have limited ability to prevent or treat CALs in immunoglobulin-resistant KD patients, as they showed the same cardioprotective effects as second IVIG infusion. Neither initial IVIG nonresponders nor patients treated with early initial IVIG with methylprednisolone pulse therapy are at a lower risk for coronary artery abnormalities [29]. A retrospective cohort study reported no difference in the prevalence of CALs between spontaneous defervescence KD patients without drug infusion and typical KD patients treated with initial IVIG [36]. Moreover, KD may continue to be associated with the development of severe aneurysms in a small percentage of patients (10%) who respond to initial IVIG treatment, and half of the children who developed a coronary artery aneurysm did so despite treatment [37, 38].

The results revealed that transient hepatomegaly was most likely associated with infliximab treatment [14, 16]. However, no hepatomegaly events occurred during a larger infliximab trial. The IVMP group reported more AEs during treatment; these events included chills, headache, hemolytic anemia, coagulopathy, hypertension, hypothermia, bradycardia, hyperglycemia, gastrointestinal bleeding, nerve palsy, and shock (Table 2). Nagakura et al. suggested that bradycardia might occur frequently during corticosteroid treatment, and bradycardia was associated with responsiveness to treatment in a cohort study [39]. However, the rate of AEs associated with IVMP infusion was significantly lower than that associated with infliximab or IVIG retreatment, which might be explained by the following two reasons. First, reporting bias may exist for methylprednisolone, as a classic anti-inflammatory drug administered to IVIG-resistant KD patients, AEs associated with methylprednisolone have been extensively reported. In contrast, infliximab is a chimeric monoclonal antibody that has been used in recent years, and there are relatively few reports of untoward effects. Therefore, the IVMP group reported more AEs than the infliximab group in our meta-analysis. Second, a portion of the reported AEs were related to immunoglobulin-resistant KD or occurred before drug administration; therefore, they may not reflect the actual difference between the IVMP and infliximab groups.

Millar et al. suggested that corticosteroid use in the acute phase of KD in patients with evolving coronary artery aneurysms might be associated with worsened aneurysms and impaired vascular remodeling [40]. According to the AHA, steroid treatment should be restricted to children in whom ≥2 IVIG infusions have been ineffective for the treatment of persistent fever [1]. To date, several trials have adopted a predicted CAL scoring system and have suggested that steroids may be beneficial in reducing coronary artery aneurysms and safe for patients with immunoglobulin-resistant KD [41, 42]. However, Song et al. revealed that 4 current scoring systems (e.g., Egami, Kobayashi, San Diego, and Formosa) had limited utility in predicting immunoglobulin-resistant KD [43], which indicated that the above trials might have exaggerated the effect of steroid treatment. Conversely, a 2013 meta-analysis showed that IVIG plus corticosteroid therapy as an initial therapy significantly reduced the risk of CALs [44]. Moreover, a recently published meta-analysis highlighted the importance of timing for the prevention of CALs when treating KD patients [45]. Briefly, according to the GRADE evidence profile, although IVMP was not more advantageous than infliximab or second IVIG with respect to cardioprotective effects or lowering the rate of treatment resistance, this treatment might have the ability to attenuate the severity of KD.

Certain laboratory parameters in KD patients are considered useful markers of inflammation that may reflect disease severity and treatment effects; such parameters include leukocyte and platelet counts, erythrocyte sedimentation rate, and the levels of hemoglobin, C-reactive protein, albumin, TNF-α, monocyte chemoattractant protein-1 (MCP-1), aspartate aminotransferase (AST) and alanine aminotransferase (ALT). Previous studies revealed that IVIG nonresponders have a higher neutrophil differential, higher C-reactive protein levels, and lower cholesterol levels than responders, and there was a high risk of CALs in patients with more severe and persistent inflammation [46, 47]. Additionally, the available data from the selected studies indicate that the anti-inflammatory effects of IVMP might be superior to those of infliximab. The variations in laboratory findings of IVIG-resistant KD patients may be beneficial for modifying treatment strategies in the future. However, due to a limited number of appropriate studies and the absence of suitable data at presentation, neither inflammatory markers nor laboratory results were analyzed in our meta-analysis. Therefore, a randomized, double-blind, multicenter, parallel-group trial should be conducted to assess IVMP versus infliximab in immunoglobulin-resistant KD patients; this study should contain a standard operation procedure (SOP) for echocardiography based on the AHA guidelines and a stratified analysis of the initial discrepant inflammation intensity between treatment groups at study entry.

Cardiovascular manifestations and complications are closely connected to morbidity and mortality associated with severe KD, during both acute illness and long-term follow-up. Early diagnosis and early IVIG infusion in incomplete KD patients could reduce the risk of CALs [48, 49]. Risk stratification allows for individualized long-term patient management regarding the frequency of follow-up and diagnostic testing, cardiovascular risk factor assessment and management, medical therapy, thrombo prophylaxis, physical activity, and reproductive counseling, which may have a considerable benefit for severe KD patients [2].

To date, because few clinical trials have assessed the efficacy of medications other than second IVIG treatment, neither the AHA nor the Research Committee of the Japanese Society of Pediatric Cardiology (RCJSPC) reached consensus on the treatment options for IVIG-resistant KD. Both the AHA and RCJSPC recommend mostly a second IVIG treatment as the best reasonable therapy in IVIG-resistant patients (AHA IIa/B; RCJSPC III/B), secondly as IVMP (AHA IIb/B; RCJSPC IIb/B), then as infliximab treatment (AHA IIb/C; RCJSPC IIb/C) [1, 26, 50]. Compared to RCJSPC, the AHA (2017) highlighted that IVMP could be considered an effective alternative to a second infusion of IVIG. Meanwhile, our meta-analysis has provided the best available evidence that infliximab, IVMP, and a second IVIG infusion showed no significant differences in the cardioprotective effect or the rate of treatment resistance, but that IVMP has advantages in antipyretic effects and a lower total rate of AEs. For this reason, our study further confirmed the potential value of IVMP treatment in IVIG-resistant KD patients. The results could be conducive for recommending an objective order of these treatment options in later studies and guidelines. In particular, considering the risk-benefit balance of IVIG [50], IVMP could exert more influence on the management of refractory KD patients in the future.

Nevertheless, this meta-analysis had several limitations. First, the use of an indirect comparison might have created differences in the clinical outcomes assessed herein. However, in the absence of sufficient head-to-head data pertaining to different treatments, an adjusted indirect comparison of the treatments in question can produce reasonable results. Some clinicians have even argued that adjusted indirect comparisons produce less bias than direct comparisons [18, 51]. Until data from direct clinical trials are available, the results of our meta-analysis represent the best available evidence. Second, similar to previous pair-wise meta-analyses, there were no detailed definitions of IVIG resistance during the observation period after drug infusion, and the body temperature for non-responsiveness was not uniformly defined in the studies included in our meta-analysis according to the guidelines of the Japanese Ministry of Health and Welfare or the AHA. The differentiation of the observation period may be attributed to the differences between medical systems and ethnicity. Furthermore, differences in the location of body temperature measurements (oral, rectal, and axillary) may have affected the analysis. Heterogeneity among the included studies in the observation period after IVMP or infliximab treatment and the body temperature indicative of IVIG resistance may have introduced potential bias. Third, potential bias may exist because of the initial discrepant inflammation intensity between treatment groups in some of the included studies. Fourth, outcomes associated with a Z score in an adjusted indirect meta-analysis are needed to better evaluate coronary artery status. Fifth, the reporting bias was minimized, as we retrieved unpublished data from gray literature. However, all the studies included in this analysis were derived from published literature, and some unpublished studies remain missing. Finally, although no significant statistical or clinical heterogeneity was observed across the included studies, potential bias still exists because the relevant literature is limited, and most of the included studies did not completely evaluate the post-retreatment incidence of coronary artery aneurysms in patients with immunoglobulin-resistant KD after a short-term follow-up. Therefore, large homogeneous and randomized clinical trials with long follow-up periods are needed, especially trials involving infliximab.

## Conclusion

Neither infliximab nor IVMP was associated with cardioprotective effects or decreases in the rate of treatment resistance compared with second IVIG infusion, and both treatments were more effective than second IVIG infusion due to their antipyretic effects. Additionally, IVMP may have an advantage due to its lower total rate of AEs associated with drug infusion. However, the results of this meta-analysis should be interpreted with caution due to the presence of potential limitations. Until data from direct clinical trials comparing infliximab with IVMP are available, our meta-analysis provides preliminary evidence for the optimal management of immunoglobulin-resistant KD patients.

## Additional file

Additional file 1: Search strategy. (DOCX 16 kb)

## Abbreviations

AEs: Adverse effects
CALs: Coronary artery lesions
IVIG: Intravenous immunoglobulin
IVMP: Intravenous pulse methylprednisolone
KD: Kawasaki disease
Non-RCTs: Non-randomized clinical trials
R: Risk ratio
RCTs: Randomized clinical trials
