# Supplementary material for: Indirect-comparison meta-analysis of treatment options for patients with refractory Kawasaki disease
Source: BMC Pediatr. 2019 May 17;19:158. doi: 10.1186/s12887-019-1504-9 (PMC6524334; doi:10.1186/s12887-019-1504-9)
Supplement: Supplementary file 1 — Search strategy. (DOCX 16 kb) [file 12887_2019_1504_MOESM1_ESM.docx]

**Pubmed:**

#12,"Search (((""Mucocutaneous Lymph Node Syndrome""[Mesh]) OR Kawasaki disease)) AND (#10)) Filters: Publication date to 2018/08/31"

#11,"Search Filters: Publication date to 2018/08/31"

#10,"Search #3 OR #4 OR #5 OR #6 OR #7 OR #8"

#9,"Search (""Mucocutaneous Lymph Node Syndrome""[Mesh]) OR Kawasaki disease"

#8,"Search IG"

#7,"Search IVGG"

#6,"Search IVIG"

#5,"Search intravenous gamma globulin"

#4,"Search intravenous immunoglobulin"

#3,"Search ""Immunoglobulins, Intravenous""[Mesh]"

#2,"Search Kawasaki disease"

#1,"Search ""Mucocutaneous Lymph Node Syndrome""[Mesh]"

**Embase:**

#11. #9 AND #10

#10. #3 OR #4 OR #5 OR #6 OR #7 OR #8

#9. #1 OR #2

#8. ig AND [1-1-1968]/sd NOT [31-08-2018]/sd

#7. ivgg AND [1-1-1968]/sd NOT [31-08-2018]/sd

#6. ivig AND [1-1-1968]/sd NOT [31-08-2018]/sd

#5. intravenous AND gamma AND globulin AND

[1-1-1968]/sd NOT [31-08-2018]/sd

#4. intravenous AND immunoglobulin AND [1-1-1968]/sd

NOT [31-08-2018]/sd

#3. 'intravenous immunoglobulin'/exp AND

[1-1-1968]/sd NOT [31-08-2018]/sd

#2. kawasaki AND disease AND [1-1-1968]/sd NOT

[31-08-2018]/sd

#1. ('mucocutaneous lymph node syndrome'/exp OR

'mucocutaneous lymph node syndrome') AND

[1-1-1968]/sd NOT [31-08-2018]/sd

**Web of Science:**

# 1 TS="Mucocutaneous Lymph Node Syndrome"

# 2 TS="Kawasaki disease"

# 3 TS="intravenous immunoglobulin"

# 4 TS="intravenous gamma globulin"

# 5 TS="IVIG"

# 6 TS="IVGG"

# 7 TS="IG"

# 8 #2 OR #1

# 9 #7 OR #6 OR #5 OR #4 OR #3

# 10 #9 AND #8

# 11 PY=1900-2018

# 12 #11 AND #10

**Cochrane Database**

1. Title Abstract Keyword (mucocutaneous lymph node syndrome)

2. Title Abstract Keyword (Kawasaki disease)

3. S1 OR S3

**ProQuest Dissertations & Theses**

#1 (intravenous immunoglobulin) OR (intravenous gamma globulin) OR IVIG OR IVGG OR IG

#2 (mucocutaneous lymph node syndrome) OR (Kawasaki disease)

#3 #1 AND #2

#4 (methylprednisolone OR infliximab)

#5 #3 AND #4

#6 1957 - 2018-08-31[Application filters]

#7 #5 AND #6
